# Sustained, Area-Wide Control of *Aedes aegypti* Using CDC Autocidal Gravid Ovitraps

**DOI:** 10.4269/ajtmh.14-0426

**Published:** 2014-12-03

**Authors:** Roberto Barrera, Manuel Amador, Verónica Acevedo, Ryan R. Hemme, Gilberto Félix

**Affiliations:** Entomology and Ecology Activity, Dengue Branch, Centers for Disease Control and Prevention, Calle Canada, San Juan, Puerto Rico

## Abstract

We have shown that the Centers for Disease Control and Prevention (CDC) autocidal gravid ovitraps (AGO trap) reduced the *Aedes aegypti* population and prevented mosquito outbreaks in southern Puerto Rico. After showing treatment efficacy for 1 year, we deployed three traps per home in an area that formerly did not have traps and in a site that served as the intervention area. Two new areas were selected as reference sites to compare the density of *Ae. aegypti* without traps. We monitored mosquitoes and weather every week in all four sites. The hypotheses were the density of *Ae. aegypti* in the former reference area converges to the low levels observed in the intervention area, and mosquito density in both areas having control traps is lower than in the new reference areas. Mosquito density in the former reference area decreased 79% and mosquito density in the new reference areas was 88% greater than in the intervention areas.

## Introduction

Dengue is the most common arboviral disease in the world, and one that has continued to increase in incidence over the last five decades.^1^ Because vector control is the only means to control dengue virus transmission currently available,^2^ the sustained increase of dengue probably reflects lack of significant impact or at the best, a partial impact of vector control without which dengue figures would be even larger. The control of dengue vectors is complicated by a lack of trained personnel and resources and by adopting reactive approaches to dengue control only during epidemics. Furthermore, many indispensable (e.g., water-storage containers) and disposable containers (e.g., trash, junk) result from urbanization in the absence of adequate public services. Population growth has increased urban complexity in mosquito habitat compartmentalization and limits the required access of vector control personnel to premises.^3^ Additional factors limiting dengue control are elevated levels of insecticide resistance and lack of evaluation of the efficacy of vector control measures.^4^

Current approaches to controlling dengue virus vectors rely on the control of immature mosquitoes or adults. Immature stages of mosquitoes are generally controlled by removing containers that can be used as larval habitats or through the application of larvicides, whereas adults are controlled by spatial spraying of pesticides.^2^ Indoor residual spraying is being applied for the focal control of *Aedes aegypti* in and around dengue cases in Australia^5^ but this technique is not commonly used elsewhere. Adulticiding techniques have limited effects on existing adult mosquitoes and does not allow for sustained control of vector populations.^6^ Aside from increased resistance to the most widely used larvicide (temephos^7^,^8^), there are two main limitations in controlling immature mosquitoes. The first of which is gaining access to the houses or properties that are producing the mosquitoes because the residents are absent or refuse entry to vector control personnel.^3^ This prevents achieving area-wide management of the vector, and mosquitoes from houses that did not receive control measures are able to recolonize habitats in adjacent properties. Access to properties can be compensated by adjusting the work schedule of vector control programs, and by creating legislation facilitating access of health inspectors to premises. The other limitation is the existence of cryptic aquatic habitats, which produce dengue virus vectors, such as storm drains, septic tanks, roof gutters, elevated water tanks, and depressions on roof tops,^9^–^15^ some of which are locally abundant and highly productive. When cryptic habitats are present, but not identified, only the containers that are visible will be treated resulting in incomplete application of control measures, which in turn virtually guarantees the failure of vector control programs.^14^ A way to determine if important cryptic aquatic habitats exist in a locality is by monitoring the impact of immature control on both immature and adult mosquito populations.^15^

Vector control programs are in great need of developing new control tools that complement the control of immature mosquitoes^16^,^17^ and better entomological surveillance tools to assess the impact and sustainability of specific or integrated control measures. There are several promising approaches to dengue virus vector control that target the adult stages of the mosquito, such as new adulticides,^18^ insecticide impregnated materials (curtains,^19^ bed nets,^20^ covers for water-storage containers,^21^ and ovitraps^17^), sticky gravid traps,^22^,^23^ auto-dissemination of insect growth regulators by contaminated (pyriproxyfen)^24^ or infected (fungi spores)^25^ adult mosquitoes, and the release of genetically modified^26^ and *Wolbachia*-infected mosquitoes.^27^,^28^

Better tools for entomological surveillance, mainly centered on tracking the adult stages of dengue virus vectors have been developed, such as a more practical electro-mechanical aspirator,^29^ active mosquito traps,^30^ and a number of passive traps for gravid females that use funnels,^31^ sticky surfaces,^32^–^34^ or insecticides.^35^ Novel tools to monitor adult mosquito populations can help estimate the impact of vector control measures when used in well-designed field experiments. Ideally, field experiments would account for the confounding effects of spatial heterogeneity and temporal changes in factors that influence the abundance and structure of mosquito populations, such as weather. Longitudinal studies are well suited to answer these questions.^36^

The current investigation is a continuation of a longitudinal study on the impact and sustainability of sticky gravid ovitraps (Centers for Disease Control and Prevention [CDC] autocidal gravid ovitrap [AGO] traps^22^) as a control tool for *Ae. aegypti*. There were two main objectives of this study. The first was to determine if the sustained reduction in the *Ae. aegypti* female populations observed during the first year of the investigation in an urban area was maintained over time. The second objective was to examine if adding intervention traps to a site that was originally used as a non-intervention reference area would succeed in lowering the population abundance of female *Ae. aegypti* to levels that have been observed in a separate intervention site. Our results confirmed that the use of three AGO traps per home produced consistent and significant reductions in the population of *Ae. aegypti* over time.

## Materials and Methods

### Study areas.

Four urban areas in southern Puerto Rico were selected as study sites. Most buildings in these areas were one story houses with patios and had reliable sanitary services, such as piped water and domestic garbage pickup (Table 1). All sites had sewerage, however in Playa and Villodas several houses still used septic tanks. Intervention area I (IA-I; La Margarita) was isolated from neighboring buildings by a 200 m stretch of vegetation, whereas intervention area II (IA-II; Villodas) was separated from other urbanized areas by ∼500 m of vegetation and roads. Isolation between urban areas was considered necessary to minimize mosquito migration from nearby areas into the intervention areas.^37^,^38^ The two reference areas (RA): Arboleda (RA-I) and Playa (RA-II) were part of a larger urban area, and they did not need to be isolated from nearby *Ae. aegypti* populations.

Meteorological stations (HOBO Data Loggers, Onset Computer Corporation, Boume, MA) were placed in the center of La Margarita, Villodas, and Arboleda to monitor air temperature, relative humidity, and rainfall. No meteorological station was placed in Playa because this location was just 200 m south of La Margarita. There is a cooler and drier season from December to March and a warmer and wetter season for the rest of the year in the study areas.^22^ The main difference in weather among study sites was the wetter conditions registered in Villodas (Table 1).

### AGO traps.

The sticky AGO trap has been previously described.^22^,^34^ It is a passive trap made out of 1) a black polyethylene pail (19 L of volume) holding 10 L of water and a 30 g hay packet to attract gravid mosquitoes, and 2) an upper trap entrance component that houses the sticky surface. The sticky glue was a non-setting, polybutylene adhesive (32UVR, Atlantic Paste & Glue Co., Inc., Brooklyn, NY), which had been successfully used in sticky traps before.^39^ A fine-mesh screen prevented mosquitoes from reaching the water, although at the same time allowed water vapor and odorants to escape through the top of the trap. The screen also prevented any adult mosquito emerging from the infusion to escape, which can happen when eggs from dead females are washed into the infusion by rains.^32^ A funnel made out of plastic screen (19.5 cm dia. × 10.5 cm high × 5.5 cm dia.) was placed at the entrance of the AGO traps that we used for mosquito elimination to reduce the entrance of domestic lizards (CDC, unpublished data). Traps were serviced every 2 months, which consisted of cleaning the outer surface of the trap and replacing the sticky board, hay packet, and replenishing with water. Previous observations indicated sustained capture rates of *Ae. aegypti* per week during the 8 weeks between trap servicing.^34^

**Figure 1.**
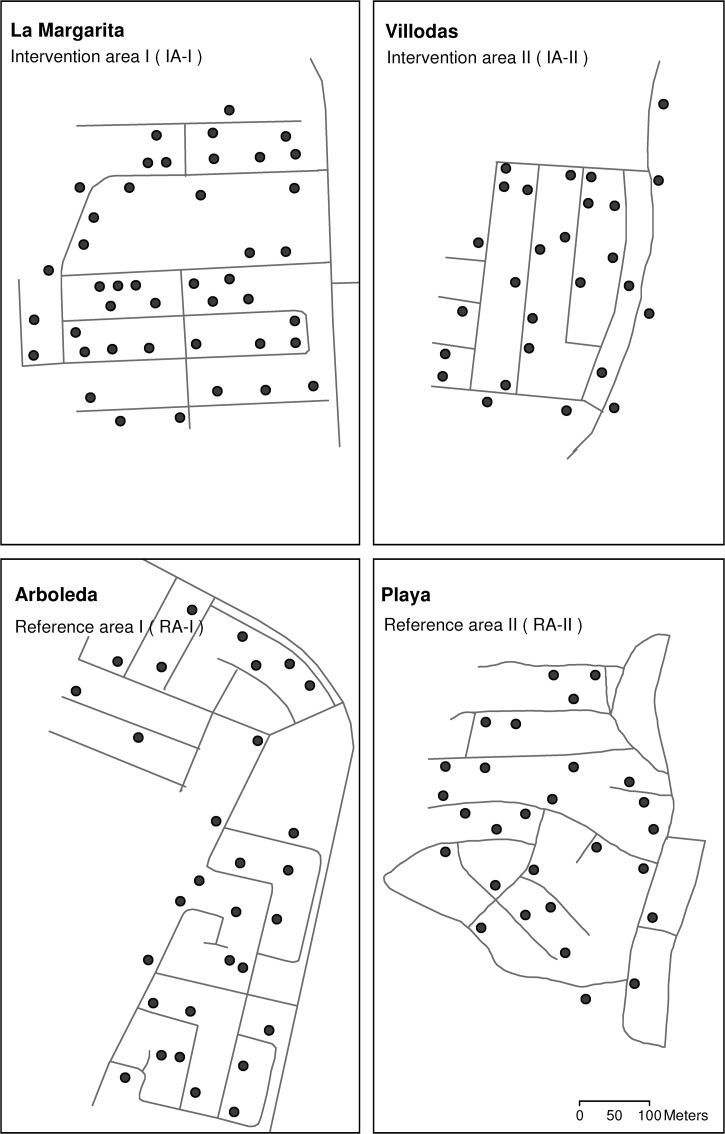
Location of sentinel autocidal gravid ovitraps (AGO traps) deployed at fixed locations to monitor the number of female *Ae. aegypti* per week in each of the four study areas.

The AGO traps can be used to monitor or control *Ae. aegypti* females. Sentinel AGO traps (SAGO traps) used for mosquito surveillance were visited once a week to remove and count all trapped mosquitoes. Traps used for mosquito control purposes in intervention areas were left in the field for 2 months and were not used for surveillance purposes. Previous research has shown that the number of female *Ae. aegypti* captured in AGO and BG-Sentinel traps were significantly correlated,^22^ therefore only SAGO traps were used for mosquito surveillance in this study. We used between 27 and 44 fixed-position SAGO traps in each study area to monitor the density of female *Ae. aegypti* per week (Table 1; Figure 1). The SAGO traps were separated from each other by a minimum of 30 m to avoid trap interactions and spatial auto-correlations.^40^ All SAGO traps in the four study sites were inspected on the same day of the week, and consisted of picking adult mosquitoes using tissue probes or forceps. Mosquitoes were identified to species and sexed by placing them on a white paper towel for better visibility. Aside from *Ae. aegypti*, *Culex quinquefasciatus* mosquitoes were regularly trapped. Extracting mosquitoes every week from SAGO traps was needed to insure that any mosquitoes present in the traps the following week was a new catch.

### Experimental design.

In a previous study, we placed three AGO intervention traps per house in 81% of the houses in La Margarita (IA-I) and used Villodas (IA-II) as a reference (without intervention traps) to compare the number of female *Ae. aegypti* every week from October 2011 to 2012.^22^ The current investigation is a continuation of that study where AGO intervention traps were placed in 85% of the houses in both localities. Thus, AGO intervention traps have been in La Margarita (IA-I) from December 2011 to February 2014 and in Villodas (IA-II) from February 2013 to 2014.

#### Impact of AGO intervention traps.

Research hypothesis 1 stated that the density of *Ae. aegypti* in Villodas (IA-II) would be significantly reduced after trap deployment. To test this hypothesis we compared the average weekly density of female *Ae. aegypti* per trap in Villodas (IA-II) before (June 2012–February 2013) and after placing the AGO intervention traps (February 2013–2014). Research hypothesis 2 stated that the density of *Ae. aegypti* in Villodas (IA-II) after placing the traps was similar to that observed in La Margarita (IA-I) where AGO intervention traps had been in place since December 2011. When placing the AGO intervention traps in Villodas (IA-II), we also implemented source reduction measures that included the removal of artificial containers, larviciding, and oviciding to reduce the availability of containers with water and temporarily reduce the population of *Ae. aegypti*. These control measures were also implemented in La Margarita (IA-I) at the beginning of the study in December of 2011.^22^

#### Comparing areas with and without AGO intervention traps.

To compare these results with the density of *Ae. aegypti* in non-intervention areas, we selected two nearby urban areas: Arboleda (Reference Area I [RA-I]) and Playa (Reference Area II [RA-II]; Table 1; Figure 1). Research hypothesis 3 was that *Ae. aegypti* density in areas with AGO intervention traps (IA-I and IA-II) was significantly lower than in the new reference areas (RA-I and RA-II). The new RAs were concurrently monitored from February 2013 to February 2014 (after placing the AGO control traps in IA-II).

### Statistical analyses.

We tested null hypothesis 1 that the density of *Ae. aegypti* females (individuals per trap per week) in the new intervention area (IA-II) was the same before and after placing three AGO traps per home. A generalized linear mixed model (GLMM) was used to test for the main effect of treatment (before, after trap placement) in Villodas (IA-II), whereas controlling for the week of sampling between trap servicing (1–8 weeks), rainfall (accumulated rainfall during the third and second weeks before sampling), relative humidity (average of 7 days before sampling), and temperature (average for 3 weeks before sampling).^41^ The distribution probability function of the dependent variable was the negative binomial with log link. The covariance structure for the repeated estimation of mosquito density per trap per week was a first-order autoregressive function. The model variables location and house ID were used as random factors to account for location bias using a covariance component identity matrix.

We tested null hypothesis 2 that after placing AGO intervention traps in Villodas (IA-II), mosquito density was going to reach similar values to those observed in La Margarita (IA-I), where a similar treatment with three AGO traps per home had been in place since December 2011. A GLMM model was used as before but the main effect tested was location (IA-I versus IA-II).

Null hypothesis 3 stated no significant differences in female *Ae. aegypti* density per week among the four study sites (IA-I, IA-II, RA-I, RA-II), or that placing three AGO intervention traps around each home in the intervention areas would not significantly affect mosquito density from February 2013 to February 2014. A GLMM was used to test for the differences in average number of female *Ae. aegypti* per trap per week where study site was the main effect. The same covariates and model specifications were used as before. All statistical analyses were performed using IBM SPSS Statistics 20 software (IBM Corporation, Armonk, NY).

## Results

### Impact of AGO intervention traps.

**Figure 2.**
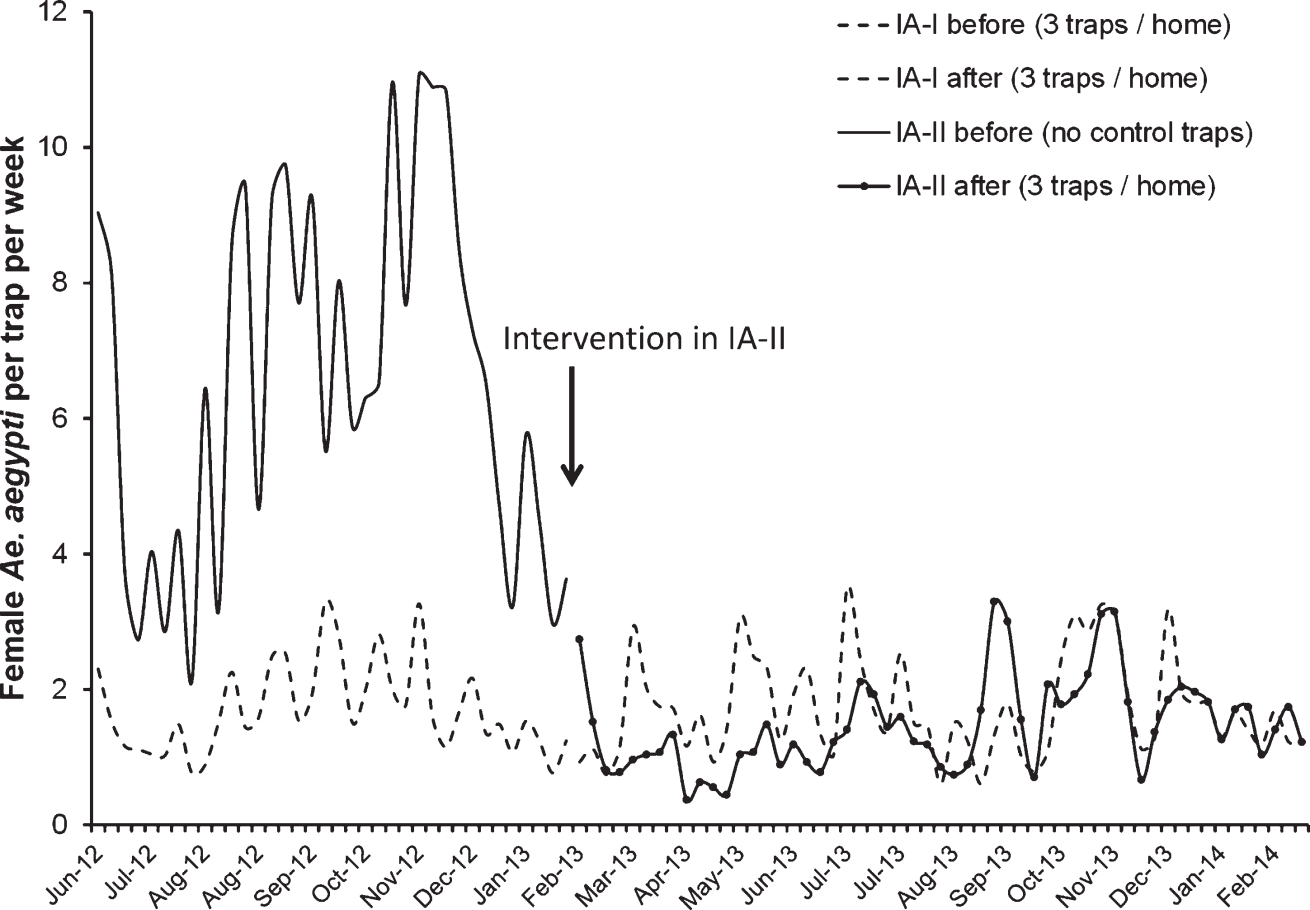
Changes in average *Ae. aegypti* females per trap per week in the new intervention area (IA-II; Villodas) after placing autocidal gravid ovitraps (AGO traps) in February 2013 and in the previous intervention area (IA-I; La Margarita) where AGO traps had been in place since December 2011.

There was an average reduction of 79% in the number of female *Ae. aegypti* per trap per week after placing the AGO intervention traps in IA-II (estimated means ± 95% confidence interval [CI]; before = 5.7; 5.0–6.6, after = 1.2; 1.0–1.4; Figure 2). The GLMM model showed significant effects of trap placement (before, after; F_1, 2425_ = 798.3, *P* < 0.001), week after trap servicing (F_7, 2425_ = 5.9, *P* < 0.001), rainfall (F_1, 2425_ = 16.3, *P* < 0.001), relative humidity (F_1, 2425_ = 47.2, *P* < 0.001), and temperature (F_1_, _2425_ = 5.2, *P* < 0.05).

Average captures in Villodas (IA-II) after placing the AGO control traps (1.2; 1.0–1.4 females per trap per week) were lower than in La Margarita during the same time period (1.6; 1.4–1.8; February 2012–2014). The GLMM model showed significant effects of site (F_1_, _3756_ = 7.9, *P* < 0.01), week after trap servicing (F_7, 3756_ = 17.8, *P* < 0.001), rainfall (F_1, 3756_ = 13.0, *P* < 0.001), and relative humidity (F_1, 3756_ = 15.5, *P* < 0.001). However, the densities of *Ae. aegypti* in both localities seemed to have converged to similar values during the last weeks of observations (Figure 2).

Average captures in IA-I were similar before (average ± 95% CI; 1.5 ± 0.2) and after (1.6 ± 0.2) the point in time when traps were placed in IA-II, indicating that the presence of AGO intervention traps in IA-I (La Margarita) consistently reduced the density of mosquitoes throughout the study (Figure 2).

**Figure 3.**
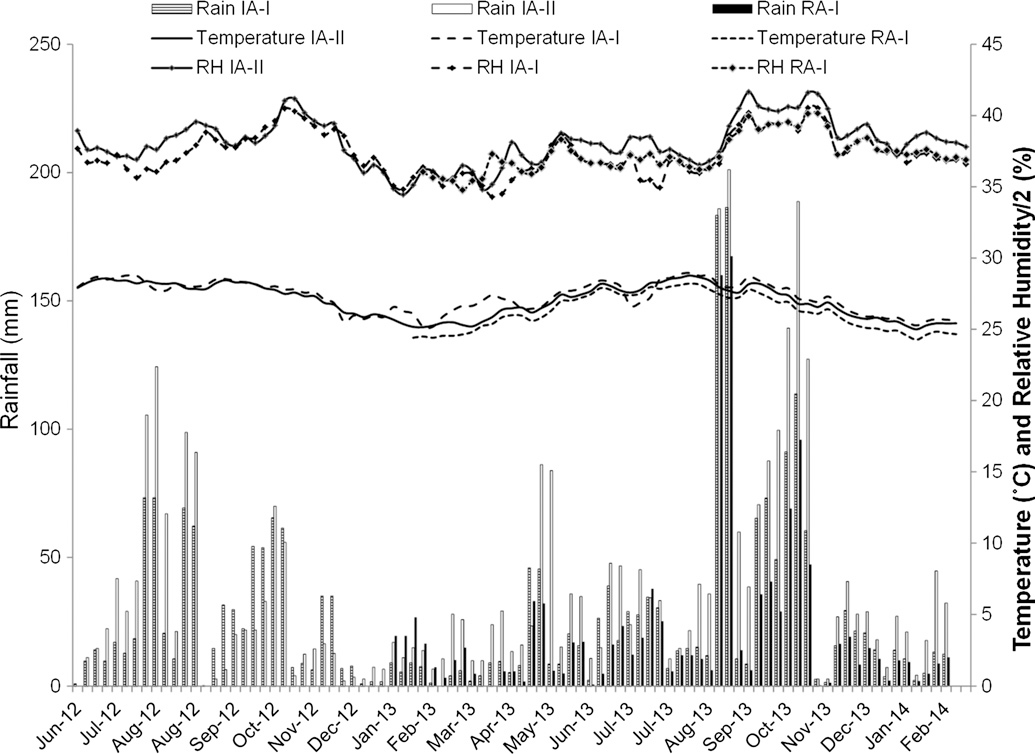
Variations in weather variables registered in three meteorological stations located in two intervention and one reference sites during the study.

Accumulated rainfall during the third and second weeks before sampling and average relative humidity during the week before sampling (Figure 3) were positively and significantly associated with the density of female *Ae. aegypti*. These two meteorological variables were positively correlated but were kept in the model because they may affect both the immature and adult stages of *Ae. aegypti*. There were marked inter-annual variations in the amount of precipitation between 2012 and 2013 (Figure 3).

**Figure 4.**
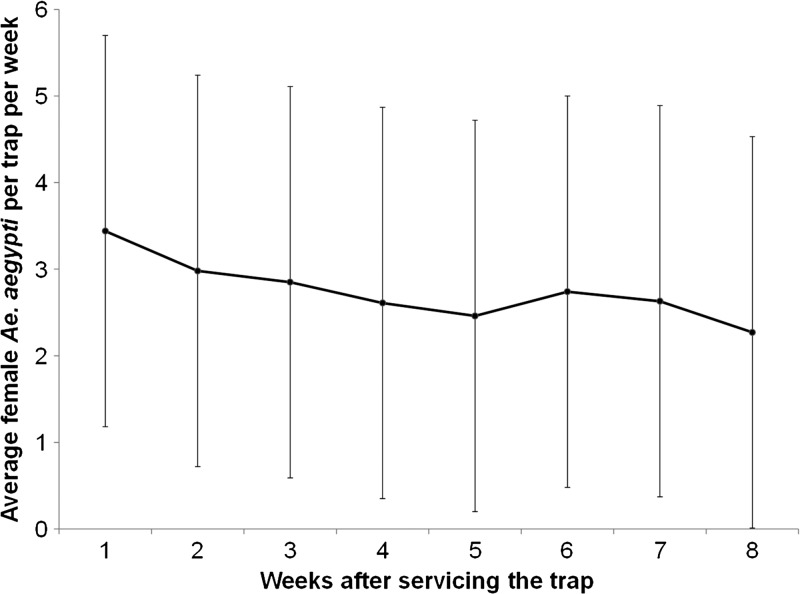
Model-estimated average numbers of *Ae. aegypti* females per trap per week in sentinel autocidal gravid ovitraps at weekly intervals (1–8) after trap servicing to observe any changes in trap efficacy over time.

Trap captures in SAGO traps were evaluated weekly after traps were serviced, which occurred every 8 weeks (Figure 4). The results indicated a slight reduction in trap efficacy over time after replenishing the traps with water, changing the sticky surface, and adding a new pack of hay the eighth week (Figure 4).

### Comparing areas with and without AGO intervention traps.

**Figure 5.**
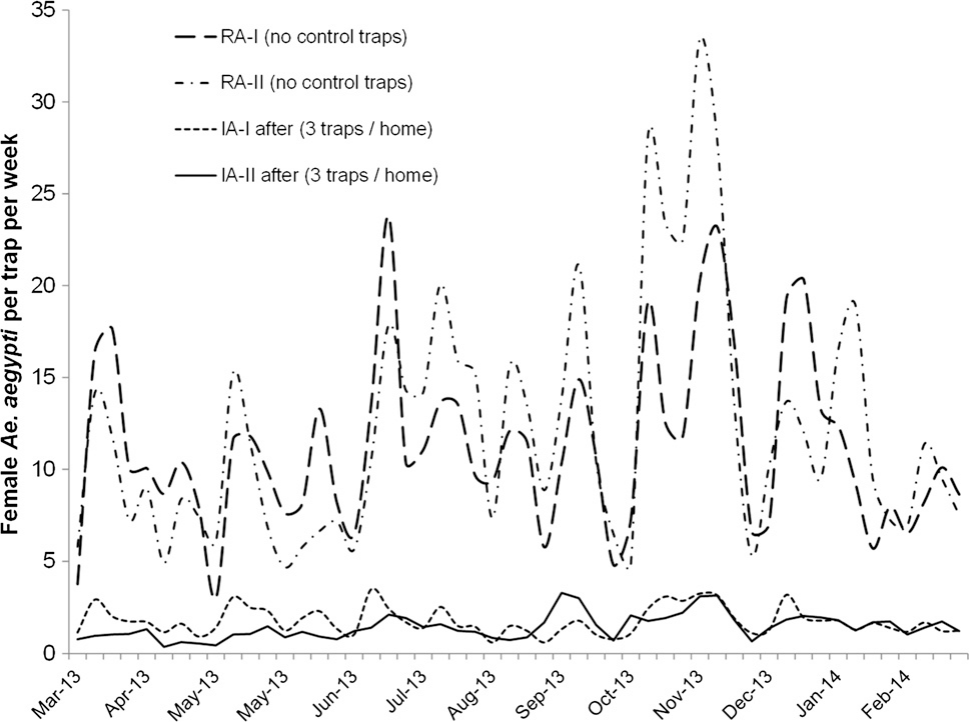
A comparison of average *Ae. aegypti* females per trap per week in two autocidal gravid ovitraps (AGO traps) intervention (IA-I, La Margarita; IA-II, Villodas) and two reference areas (RA-I, Arboleda; RA-II, Playa) in southern Puerto Rico from February 2013 to 2014.

The average number of female *Ae. aegypti* per trap per week significantly varied among sites (Figure 5; F_3, 7211_ = 11.0, *P* < 0.001), sampling weeks after trap servicing (F_7, 7211_ = 91.4, *P* < 0.001), rainfall (F_1, 7211_ = 63.9, *P* < 0.001), and relative humidity (F_1, 7211_ = 12.6, *P* < 0.001). The average numbers of female *Ae. aegypti* per trap per week (model estimated means; 95% CI) in the reference areas RA-I (10.4; 4.9–20.9) and RA-II (12.9; 6.2–26.6) were greater than in the intervention areas IA-I (1.5; 0.7–3.1) and IA-II (1.3; 0.6–2.6). Thus, areas with AGO intervention traps had 88% fewer mosquitoes than nearby reference areas. It was observed that *Ae. aegypti* females sharply increased after rains (Figure 3) in the reference areas, whereas the magnitude of the increase was much less in the intervention areas (Figure 5).

Compared with females, fewer male *Ae. aegypti* were trapped from February 2013 to February 2014 in the SAGO traps: IA-I (females = 4,135; males = 249), IA-II (females = 2,260; males = 167), RA-I (females = 18,209; males = 826), and RA-II (females = 23,143; males = 3,118).

## Discussion

In a previous study we showed that placing three AGO traps per house (yard, garden) caused a significant and stable reduction (60%) in the number of female *Ae. aegypti* in an intervention area as compared with a reference area without intervention traps.^22^ In this investigation the same number of traps per home were placed in a former reference area (Villodas) and the reduction in *Ae. aegypti* was compared before and after trap placement. The hypothesis stated that the density of mosquitoes in Villodas (IA-II) would be reduced to the low and stable counts that were observed in La Margarita (IA-I). Our results supported this hypothesis as the population of *Ae. aegypti* were significantly reduced by 79%. To compare these results with the naturally occurring density of this mosquito in non-intervention areas, we conducted concurrent surveillance in two nearby reference areas. Average mosquito density in both reference areas was 88% greater than in intervention areas. Thus, the results of this ongoing longitudinal study confirmed that AGO traps can substantially decrease the population density of *Ae. aegypti*.

Sticky AGO traps are effective at eliminating gravid *Ae. aegypti* females and act as population sinks for both reproductive adults and egg stages. We have previously shown that the number of gravid females in AGO traps correlated with the number of eggs in paired ovitraps^34^; a result that has also been observed in sticky traps with *Ae. albopictus*.^33^ This indicates that the low and stable counts observed in both intervention areas are most likely the result of a reduction in the number of *Ae. aegypti* eggs going into available water-filled containers over time. It was noted that the number of female mosquitoes in the intervention areas fluctuated within narrow limits but responded to an increase in rainfall accumulation, although mosquito counts never increased to produce the larger mosquito populations observed in the reference areas. This study also adds evidence that rainfall is a main driver of *Ae. aegypti* populations in Puerto Rico.^41^,^42^ Temperature tended to decrease after abundant rains as a result of increased cloudiness. Relative humidity increased after abundant rains and was significantly associated with *Ae. aegypti* females per trap, however the effect of humidity could not be distinguished from the effect of rainfall because both variables were highly correlated.

The fact that AGO traps are serviced only every 2 months is advantageous in comparison with smaller sticky gravid traps or insecticidal ovitraps that require weekly or monthly maintenance. We have previously shown that AGO traps have a sustained capacity to capture gravid females throughout an 8-week period.^43^ However, we were able to take advantage of this more exhaustive temporal study to explore trap performance over time. The results indicated that the week after servicing the trap was a significant cofactor explaining trap captures. There was a tendency to capture fewer specimens in time, but these variations did not justify servicing the traps at shorter intervals. These results applied to the SAGO traps that were checked weekly, as there were opportunities to correct any problems (e.g., objects blocking the trap entrance). This was not done for the intervention traps, which have not been assessed for capture efficiency after being serviced.

The use of AGO traps requires ample participation of the community. For example, most houses (81–85%) in each intervention area have had three traps in their yards since December 2011 in La Margarita and since February 2013 in Villodas. Permission to enter properties for trap servicing needs to be requested every 2 months, however many residents have authorized our personnel to enter their yards for this purpose even in their absence. Furthermore, to reach the coverage required for servicing most of the traps, we have adjusted our field work schedule to include weekends and after working hours to increase the likelihood that technicians would find residents at home.^3^

Future studies using AGO traps should evaluate if the reduction of mosquito populations shown in these studies would be sufficient to prevent dengue virus transmission. The elimination of gravid *Ae. aegypti* females has the added advantage of targeting this vector at the stage in which they can transmit arboviruses; that is, after having taken infectious blood meals. Another logical next step would be combining the use of AGO traps with other vector control methods, within an integrated vector management program. The AGO traps are compatible with other control methods such as source reduction and larviciding, space or residual spraying of insecticides, insecticide-impregnated materials, and the release of genetically modified or *Wolbachia*-infected males.

There is a variety of traps devised to capture gravid *Ae. aegypti* females, such as sticky,^32^,^44^–^48^ insecticidal,^23^ and mechanical^49^ traps but they are not commonly used in vector control programs. Gravid traps have been used within an integrated approach to control *Ae. aegypti* around dengue cases in Australia and Singapore,^50^,^51^ but their efficacy needs to be assessed.^52^ Ovitraps baited with water and *Bacillus thuringiensis israelensis* (Bti) were used as part of an integrated control intervention in two cities in Brazil.^53^ Several field tests using insecticidal gravid traps have shown a lack of consistent results on their impact on *Ae. aegypti* populations.^52^,^54^,^55^ One factor that works against the efficacy of gravid traps and ovitraps is the presence of naturally occurring containers that function as refuges for the reproductive population, as opposed to the sink effect of the traps. Thus, it is expected that the impact of traps targeting gravid females or their eggs would be augmented by removing competing containers and controlling the immature stages of *Ae. aegypti*^49^; a task that can be complicated by the presence of highly productive, cryptic aquatic habitats.

## Figures and Tables

**Table 1:** Description of the four study sites in southern Puerto Rico where three AGO control traps were installed per house in two intervention areas and were compared with two reference areas that did not have control traps

Location name	Type of experimental unit	Geographic coordinates	Elevation (m)	Weather (February 2013/2014): temperature (°C), relative humidity (%), and rainfall (mm)	No. buildings in study area	Area (ha.)	No. sentinel AGO traps	No. control AGO traps
La Margarita	Intervention area I (IA-I)	17° 58′ 18″ N; 66° 18′ 10″ W	3	27.2*	327	18	44 (monitored from June 2012 to February 2014)	793 (since December 2011)
				74.4				
				745				
Villodas	Intervention area II (IA-II)	17° 58′ 13″ N; 66° 10′ 48″ W	20	26.8	241	11	27 (monitored from June 2012 to February 2014)	570 (since February 2013)
				76.3				
				1,177				
Arboleda	Reference area I (RA-I)	17° 58′ 46″ N; 66° 17′ 23″ W	10	26.2	398	21	30 (monitored from February 2013 to February 2014)	0
				74.6				
				595				
Playa	Reference area II (RA-II)	17° 57′ 59″ N; 66° 18′ 10″ W	1	27.2*	269	17	28 (monitored from February 2013 to February 2014)	0
				74.4				
				745				

* The same meteorological station was used because of the proximity of these two areas.

AGO = autocidal gravid ovitrap.
